# In vitro surface and color changes of tooth-colored restorative materials after sport and energy drink cyclic immersions

**DOI:** 10.1186/s12903-022-02624-1

**Published:** 2022-12-09

**Authors:** Saijai Tanthanuch, Boonlert Kukiattrakoon, Thanyathorn Thongsroi, Premkamol Saesaw, Naphat Pongpaiboon, Supharadee Saewong

**Affiliations:** Department of Conservative Dentistry, Faculty of Dentistry, Prince of Songkla University, Hat Yai, Songkhla Thailand

**Keywords:** Color, Energy drink, Sport drink, Surface hardness, Surface roughness

## Abstract

**Background:**

There has not been any research conducted on surface properties and color changes from sport and energy drinks on bulk-fill resin composite, nanohybrid resin composite and glass ionomer restorative material. The aim of this in vitro study was to investigate the effect of sport and energy drinks on surface hardness, roughness and color changes of bulk-fill resin composite, nanohybrid resin composite and glass ionomer restorative material, and to also evaluate the acidity and titratable acidity of the drinks.

**Methods:**

One hundred and forty-seven specimens of each tooth-colored restorative material were prepared in a polytetrafluoroethylene mold (10 mm. in diameter and 2 mm. in thickness). Before immersion, baseline data of hardness, roughness, and color value were recorded. Each material was divided into 3 groups for sport drink, energy drink, and deionized water (serving as a control). The specimens were immersed in a storage agent for 5 s, then in artificial saliva for 5 s alternately for 24 cycles, and then stored in artificial saliva for 24 h. The immersion cycle was repeated for 14 days and hardness, roughness and color values were measured at 7 and 14 days.

**Results:**

After immersion, the glass ionomer restorative material had statistically less hardness, more roughness and more color changes than the others (*P* < 0.05). Energy drink groups statistically caused more surface and color changes than sport drink groups (*P* < 0.05).

**Conclusion:**

Sport and energy drinks affected hardness, roughness, and color changes in all the tooth-colored restorative materials evaluated.

## Background

Tooth-colored restorative materials are widely used and have been developed as various types including glass ionomer restorative material and resin composite. Each type of material has different properties, advantages and disadvantages [1]. Glass ionomer restorative material is widely used in dentistry because it can be bonded to teeth by chemical bonds, release fluoride, and has good physical properties and biocompatibility [2–4]. However, it still has some disadvantages such as being highly sensitive to moisture during the initial curing process, and having low tensile strength, flexural strength, fracture toughness, and wear resistance [2]. Resin composites are more widely used nowadays instead of amalgam [5–8] due to the need for more aesthetics, to preserve dentin and because it has similar mechanical properties with natural teeth [9]. Resin composites are commonly composed of a polymeric matrix and inorganic fillers [10–12], and strong bonding between matrix and fillers are enhanced by coupling agents [13]. A polymeric matrix is created from base monomer, diluents, photo initiator, accelerator, and coupling agent [10]. Nano titanium oxide (nTiO_2_), alumina (Al_2_O_3_), and hydroxyapatite are examples of inorganic fillers [10, 13]. An important improvement in the properties of resin composite is improvements of the filler with nanotechnology, resulting in a nano-resin composite [14–17].

Nano-resin composites have many good properties including improved mechanical properties, looks more beautiful, bonds better to teeth, and has less wear [5, 6]. However, resin composite still has problems with polymerization shrinkage that form gaps at the edges of the material. This increases the risk of recurrent caries. Although incremental filling techniques can reduce polymerization shrinkage, this takes much time to restore and can cause errors during each layer of restoration, especially in deep cavities, including the formation of air bubble contamination between the layers of material [18]. This led to the development of bulk-fill resin composite to address these disadvantages using polymerization modulators technology which allocate flexibility and an optimized network structure during polymerization [18]. The modulation of the polymerization reaction occurred from utilizing stress-relieving monomers, more reactive photoinitiators, as well as merging of different types of fillers such as pre-polymer particles and fiberglass rod segments [19]. Bulk-fill resin composite has distinctive properties that are different from conventional resin composites including having greater translucency and more reactive photoinitiators that can be polymerized to a depth of 4 to 5 mm and can be restored by a single filling. In addition, bulk-fill resin composite contains monomers that act as modulators of the polymerization reaction providing low polymerization shrinkage [19].

Sport and energy drinks are flavored beverages among athlete consumers. Sport drinks are flavored beverages containing carbohydrates, minerals, vitamins, and electrolytes such as sodium, potassium, calcium, and magnesium, etc. Sport drinks help to strengthen efficiency while exercising. It also replaces water and electrolytes which are lost in the form of sweat after exercise [20]. In terms of sport drinks, it is popular among athletes to quench thirst, increase energy and improve athletic performance [21]. Energy drinks have caffeine, guarana, taurine, glucuronolactone, and vitamin B. Caffeine stimulates the central nervous system resulting in refreshing the body and reducing fatigue [22].

At present, consumption of energy drinks especially among consumers aged 18–35 years, has increased [23]. Their main reason for consuming energy drinks is to reduce drowsiness, increase energy, maintain alertness while studying or driving, and to reduce hangovers [24]. However, sport and energy drinks are acidic because sport drinks contain citric acid and malic acid as their constituents [25]. Energy drinks also contain citric acid [24], which is the constituent resulting in a low pH [26]. A previous study showed that sport drinks have a pH of 3.02 ± 0.02, while energy drinks are in the range of 1.52–3.20 [27], which is in the range that can cause tooth decalcification [28]. They can also cause degradation of restorative materials [29–31] by causing extrusion of fillers from the resin matrix and result in increased surface roughness [32]. In addition, sport and energy drinks caused a significant reduction in surface hardness of the restoration material [33–35]. Energy drinks also contain synthetic colors which cause staining on the tooth-colored restorative material by adsorption and absorption procedures [36]. Adsorption stains on the material surface may be removed by polishing, while absorption stains are difficult to remove without removing the old filling material [37].

Previous studies have researched regarding either hardness or roughness or color stability on resin composite or glass ionomer restorative material [33, 34, 38–45]. Unfortunately, there has not been any research conducted on surface properties and color changes from sport and energy drinks on bulk-fill resin composite, nanohybrid resin composite and glass ionomer restorative materials. Therefore, the purpose of this study was to investigate the effect of sport and energy drinks on surface hardness, surface roughness and color changes of bulk-fill resin composite, nanohybrid resin composite and glass ionomer restorative material. The null hypothesis was that the surface hardness, surface roughness and color of bulk-fill resin composite, nanohybrid resin composite and glass ionomer restorative material would not change after immersion in sport and energy drinks.

## Methods

### Specimen preparations

Tooth colored filling materials evaluated in this study and their composition are shown in Table 1. A sample size of this study was calculated using a sample size calculation program (G*Power version 3.1.9.5, Heinrich-Heine-Universität Düsseldorf, Düsseldorf, Northrhine-Westphalia, Germany [46]) where α = 0.05, the power = 0.8 and the mean and standard deviation were taken from a previous study [34]. At least 10 samples were assigned for each group.

**Table 1:** Resin composites and glass ionomer restorative material used in this study

Material	Trade name (manufacturer)	Composition		Average particle size (µm)
		Matrix/aqueous/acid	Filler/powder	
Bulk-fill resin composite	Filtek One Bulk Fill Posterior Restorative (3 M ESPE, St.Paul, MN, USA)	AUDMA (1–10% by Wt.), UDMA (< 10% by Wt.), DDDMA (1–10% by Wt.)	Silica, Zirconia, Zirconia/Silica cluster, Ytterbium- trifluoride (YbF3). The total filler load is 76.5% by Wt (58.5% by volume)	Silica 0.02, Zirconia 0.004–0.011, Zirconia/silica cluster (comprised of 0.02 Silica and 0.004 to 0.011 Zirconia particles), 0.1 Ytterbium trifluoride (YbF3)
Nanohybrid resin composite	Premise (Kerr, Orange, CA, USA)	Bis-EMA, UDMA, TEGDMA*	Prepolymerized filler, Barium glass. The total filler load is 84% by Wt. (69% by volume)	Prepolymerized filler, Barium glass filler 0.4
Glass ionomer restorative material	Ketac Universal (3 M ESPE, St.Paul, MN, USA)	Water (40–60% by Wt.), Copolymer of acrylic acid–maleic acid (30–50% by Wt.), Tartaric acid (1–10% by Wt.)	Oxide glass (96–99% by Wt.)	No greater than approximately 2.8 µm in size

*AUDMA* Aromatic urethane dimethacrylate, *UDMA* urethane dimethacrylate, *DDDMA* 1,12-dodecanediol dimethacrylate, *Bis-EMA* ethoxylated bisphenol-A dimethacrylate, *TEGDMA* triethyleneglycol dimethacrylate

*Percent by weight not available

Five hundred and forty-one disc specimens with a diameter of 10 mm and 2 mm in thickness were prepared in a polytetrafluoroethylene mold, providing 147 disc specimens for each material. Each material was filled in the mold and the mold was placed between a bottom glass slip, a mylar matrix strip and another top glass slip. A 20 N stainless steel weight was then placed over to eliminate excess material and acquire a smooth top surface. The material was polymerized using a LED light curing unit (Elipar DeepCure-S LED Curing Light, 3 M ESPE, St.Paul, MN, USA) for 20 s. To ensure a complete polymerization, the light intensity (1,422.15 ± 3.81 mW/cm^2^) was confirmed with a measuring device (Cure Rite, L.D. Caulk, Milford, DE, USA). After complete polymerization, the top and bottom glass slips and the mylar strip were removed. No polishing procedure was necessary.

### The pH and titratable acidity measurements

The sport and energy drinks tested and their compositions in this study are shown in Table 2. The pH of each drink was measured 5 times with a pH meter (ORION900A, Orion Research, Boston, MA, USA), and the mean measured values were calculated and recorded [47].

**Table 2:** Sport and energy drinks used in this study

Beverage	Trade name	Composition	Manufacturer
Sport drink	Sponsor (250 ml)	Sucrose 17.5 g, Dextrose 10 g, Sodium chloride 0.325 g, Potassium chloride 0.075 g	T.C. Pharmaceutical Industries, Bang Bon, Bangkok, Thailand
Energy drink	M-150 (150 ml)	Sucrose 25 g, Taurine 0.8 g, Caffeine 50 mg, Inositol 50 mg, Niacinamide 20 mg, Pantothenal 5 mg, VitaminB6 5 mg, Vitamin B12 10 mcg, Citric Acid, Preservative:INS21, Synthetic Colors: INS102, INS110, Acidic buffer: INS330	Osotspa Public, Bangkapi, Bangkok, Thailand

Consequently, each drink was titrated with a 0.1 normality sodium hydroxide solution (0.1 N NaOH) by adding NaOH and measuring the pH of the drink until the drink achieved a pH of 5.5, 7 and 10, respectively. The cumulative volume of NaOH solution used for reaching each pH was recorded. Ten repetitions of titration of each drink were performed and calculated to receive a mean value [26, 47].

### Specimen immersions and testing

One hundred and forty-seven specimens of each material were divided into 3 groups with 48 specimens for each group; deionized water (serving as a control), sport drink, and energy drink groups. The remaining 3 specimens were conducted for surface micromorphology examination (before immersion). Each group was divided into 3 subgroups (16 specimens per group) for surface microhardness, surface roughness, and color measurements. Surface micromorphology examination after 7 and 14 days of the immersion period (3 specimens per period) of each subgroup were also performed.

Before immersion, the surface microhardness, surface roughness, and color values of each specimen were measured. The specimens were then alternately immersed in 25 mL of the storage agent for 5 s and 25 mL of artificial saliva for 5 s and 24 cycles [48]. Subsequently, each specimen was immersed in artificial saliva for 24 h at room temperature (approximately 25ºC). This experimental cycle was repeated for 14 days. Surface microhardness, surface roughness, and the color of each specimen were measured at 7 and 14 days [47, 48].

### Surface hardness measurements

The specimens were subjected to surface hardness (VHN) measurement with a Vickers microhardness tester (model HM-211, Mitutoyo, Kanagawa, Japan) using a 0.3 µm rectangular-based pyramidal stylus with a force of 0.5 N for 10 s, measured in 5 locations and then a mean value for each specimen was calculated. Specimens were measured for surface hardness before and after immersion at 7 and 14 days.

### Surface roughness measurements

Surface roughness measurement was performed using a profilometer (model SE2300, Surfcorder, Kosaka Laboratory, Tokyo, Japan). The 5 µm diameter stylus was moved over a distance of 2 mm with 4 mN force at a speed of 0.5 m/s with a cut off (λc) value at 0.25 mm, which was repeated 5 times for each specimen. The mean Ra (the arithmetical average of surface heights) value of each specimen was calculated before and after immersion at 7 and 14 days.

### Color measurements

The Commission Internationale de l’Eclairege L*a*b* (CIELAB) color of each specimen was assessed using a spectrophotometer (ColorQuest XE, Hunter Associates Laboratory Inc., Reston, VA, USA). Whereas L* designates the lightness of the color measured from black (L* = 0) to white (L* = 100), a* reveals the color in the red (a* > 0) and green (a* < 0) dimension, and b* clarifies the color in the yellow (b* > 0) and blue (b* < 0) dimension. Five repetitions of each specimen were performed and the mean L*, a*, and b* values were analyzed. Total color change (ΔE*) was calculated using the following equation: ΔE* = ([ΔL*]^2^ + [Δa*]^2^ + [Δb*]^2^)1/2. The mean difference of ΔE* values between the baseline and after immersion at day 7 and 14 for each group was evaluated. The consequent color change was explained as follows: if ΔE* is less than 1, it reveals that it is identical to the human eye; an ΔE* value of less than 3.3 denotes that it can be recognized by the operator’s eye but is considered clinically acceptable; and ΔE* values of ​​greater than 3.3 demonstrates that they can be perceived by non-experts’ eyes, and consequently, clinically not acceptable [49].

### Surface micromorphological examinations

Before immersion, three specimens of each nanohybrid resin composite, bulk-fill resin composite resin and glass ionomer restorative material were examined with a scanning electron microscope (JSM 5200, JEOL, Tokyo, Japan) at ×300 magnification. Subsequently after 7 and 14 days of the immersion period, the specimens from each group were also examined using the same method.

### Statistical analysis

The data obtained were subjected to statistical analysis and tested for normal distribution using Shapiro–Wilk’s test. The mean values for surface hardness, roughness, and color changes were conducted to two-way repeated ANOVA and Tukey’s Honestly Significant Difference (HSD) for multiple comparisons at a significance level of 0.05.

## Results

The mean pH and titratable acidity of sport and energy drinks are shown in Table 3. The mean pHs of both drinks were acidic. The mean pH of a sport drink (Sponsor) was higher than an energy drink (M-150). For titratable acidity, an energy drink (M-150) used more volume of NaOH solution than a sport drink (Sponsor) for titration to pH 5.5, 7 and 10.

**Table 3:** The mean pH (± SD) and titratable acidity (volume of NaOH (mL) to bring the pH to 5.5, 7.0 and 10.0) in sport and energy drinks tested

Sport and energy drink	Mean pH ± SD	Cumulative volume of NaOH used to titrate to each pH (mL)		
		5.5	7	10
Sponsor	3.54 ± 0.01	2.46 ± 0.22	2.56 ± 0.05	3.73 ± 2.69
M-150	3.43 ± 0.04	10.90 ± 0.4	13.05 ± 0.45	16.25 ± 0.52

The surface hardness, roughness, and color changes of tooth-colored restorative specimens before and after drink immersion are shown in Table 4–6. For surface hardness values in Table 4, before immersion, glass ionomer restorative materials (Ketac Universal) had significantly greater surface hardness values than the others (*P* = 0.01). However, after immersion periods, Ketac Universal had a significantly greater decrease in surface hardness values (*P* = 0.01). For surface roughness values in Table 5, before immersion, there was no significant differences in surface roughness among the materials tested (*P* = 0.68). After 14 days of immersion, Ketac Universal significantly presented the greatest roughness (*P* = 0.01). In Table 6, after immersion periods, Ketac Universal significantly presented the greatest color changes (*P* = 0.01). In general, the energy drink (M-150) caused a significant decrease in surface hardness and an increase in surface roughness and color when compared to a sport drink (Sponsor) (*P* < 0.05). The glass ionomer restorative material (Ketac Universal) significantly decreased in surface hardness and increased in surface roughness and color more than the other restorative materials tested (*P* < 0.05).

**Table 4:** The mean surface hardness (± SD) of materials tested immersed in sport and energy drinks at different times

Material	Sport and energy drink	Mean surface hardness (kg/mm^2^) ± SD at different times (day)		
		Before immersion	7 days	14 days
Filtek One	Deionized water	69.58 ± 0.23^C^	68.78 ± 0.28^a,A^	68.01 ± 0.21^a,A^
Bulk Fill	Sponsor	69.59 ± 0.57^C^	65.24 ± 0.67*^,b,B^	63.95 ± 0.36*^,b,B^
	M-150	69.65 ± 0.35^C^	62.93 ± 0.36*^,c,C^	57.67 ± 0.45*^,c,C^
Premise	Deionized water	60.67 ± 0.62^B^	59.87 ± 0.67^a,A^	59.64 ± 0.65^a,A^
	Sponsor	60.76 ± 0.48^B^	51.81 ± 0.64*^,b,D^	45.46 ± 0.52*^,b,D^
	M-150	60.68 ± 0.49^B^	49.01 ± 0.51*^,c,E^	41.42 ± 0.34*^,c,E^
Ketac	Deionized water	96.69 ± 0.42^A^	95.94 ± 0.43^a,A^	95.89 ± 0.56^a,A^
Universal	Sponsor	96.71 ± 0.38^A^	81.96 ± 0.41*^,b,F^	71.32 ± 0.45*^,b,F^
	M-150	96.82 ± 0.26^A^	76.73 ± 0.54*^,c,G^	60.92 ± 0.38*^,c,G^

*Indicates statistically significant difference (in rows) from before immersion (*P* < 0.05)

^a−c^Indicates statistically significant difference (in columns) among the three storage agents for each material (*P* < 0.05)

^A−G^Indicates statistically significant difference (in columns) among the three storage agents and three materials (*P* < 0.05)

**Table 5:** The mean surface roughness (± SD) of materials tested immersed in sport and energy drinks at different times

Materials	Storage agents	Mean surface roughness (μm) ± SD at different times (day)		
		Before immersion	7 days	14 days
Filtek One Bulk	Deionized water	0.01 ± 0.01	0.02 ± 0.01^a,A^	0.02 ± 0.01^a,A^
Fill	Sponsor	0.01 ± 0.01	0.07 ± 0.04*^, b,B^	0.12 ± 0.03*^,b,B^
	M-150	0.01 ± 0.01	0.16 ± 0.01*^,c,C^	0.18 ± 0.02*^,c,C^
Premise	Deionized water	0.01 ± 0.01	0.02 ± 0.01^a,A^	0.02 ± 0.01^a,A^
	Sponsor	0.01 ± 0.01	0.19 ± 0.02*^,b,D^	0.22 ± 0.02*^,b,D^
	M-150	0.01 ± 0.01	0.30 ± 0.02*^,c,E^	0.33 ± 0.02*^,c,E^
Ketac Universal	Deionized water	0.01 ± 0.01	0.01 ± 0.02^c,A^	0.01 ± 0.02^a,A^
	Sponsor	0.02 ± 0.02	0.33 ± 0.02*^,b,F^	0.36 ± 0.02*^,b,F^
	M-150	0.02 ± 0.02	0.29 ± 0.08*^,c,G^	0.82 ± 0.02*^,c,G^

*Indicates statistically significant difference (in rows) from before immersion (*P* < 0.05)

^a−c^Indicates statistically significant difference (in columns) among the three storage agents for each material (*P* < 0.05)

^A−G^Indicates statistically significant difference (in columns) among the three storage agents and three materials (*P* < 0.05)

**Table 6:** The mean difference of color change (∆E* ± SD) of materials tested immersed in sport and energy drinks at different times

Material	Storage agent	Mean difference of color change ± SD at different times (day)		
		Before—7 days	7–14 days	Before—14 days
Filtek One Bulk	Deionized water	1.18 ± 0.07^a,A^	0.98 ± 0.07^a,A^	1.35 ± 0.08^a,A^
Fill	Sponsor	1.80 ± 0.02^b,C^	1.07 ± 0.09^b,C^	2.30 ± 0.03^b,C^
	M-150	1.44 ± 0.05^c,B^	0.73 ± 0.05^c,B^	2.01 ± 0.09^c,B^
Premise	Deionized water	1.19 ± 0.03^a,A^	0.98 ± 0.05^a,A^	1.34 ± 0.04^a,A^
	Sponsor	2.15 ± 0.13^b,E^	1.31 ± 0.01^b,E^	2.71 ± 0.02^b,E^
	M-150	2.04 ± 0.06^c,D^	0.86 ± 0.05^c,D^	2.57 ± 0.06^c,D^
Ketac Universal	Deionized water	1.17 ± 0.05^a,A^	0.97 ± 0.06^a,A^	1.34 ± 0.05^a,A^
	Sponsor	3.16 ± 0.07^b,G^	1.19 ± 0.05^b,G^	4.59 ± 0.06^b,G^
	M-150	4.17 ± 0.06^c,F^	2.16 ± 0.07^c,F^	5.52 ± 0.04^c,F^

*Indicates statistically significant difference (in rows) from before immersion (*P* < 0.05)

^a−e^Indicates statistically significant difference (in columns) among the three storage agents for each material (*P* < 0.05)

^A−M^Indicates statistically significant difference (in columns) among the three storage agents and three materials (*P* < 0.05)

**Fig. 1:**
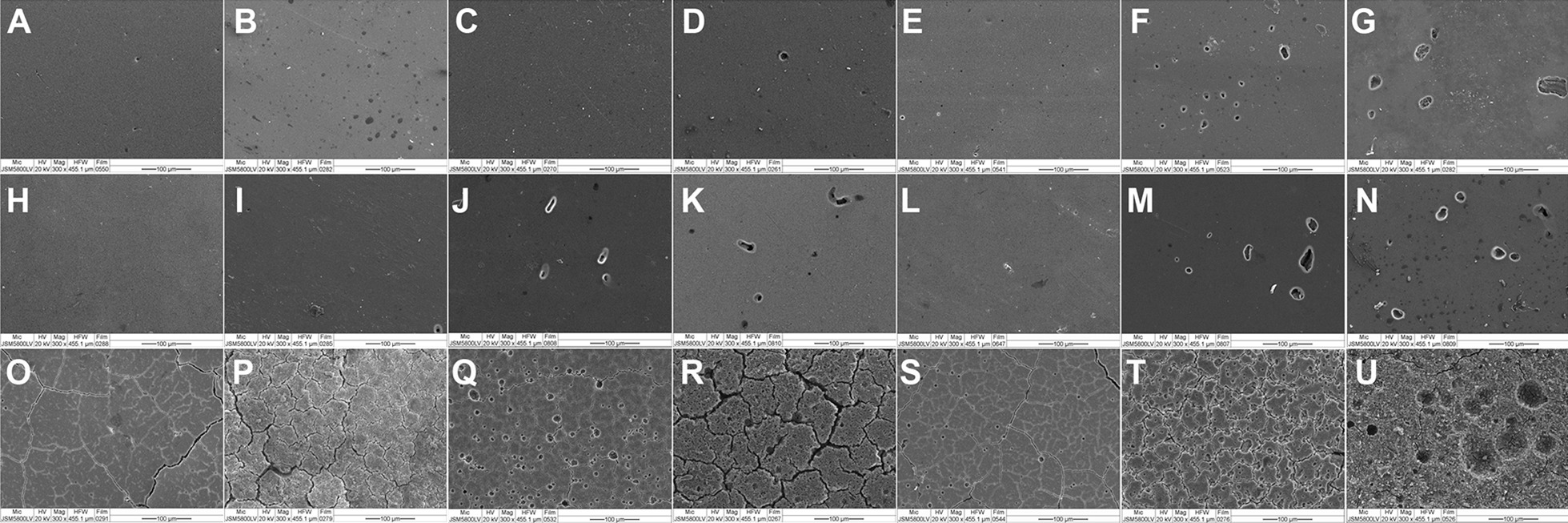
SEM photomicrographs of tooth-colored restorative material (×300). Before immersion: **A** Filtek one bulk fill posterior restorative, **H** premise, and **O** Ketac Universal. After 7 days immersion: **B** Filtek one bulk fill posterior restorative immersed in deionized water, **C** sponsor, and **D** M-150; **I** premise immersed in deionized water, **J** sponsor, and **K** M-150; **P** Ketac Universal immersed in deionized water, **Q** sponsor, and **R** M-150. After 14 days immersion: **E** Filtek one bulk fill posterior restorative immersed in deionized water, **F** sponsor, and **G** M-150; **L** premise immersed in deionized water, **M** sponsor, and **N** M-150; **S** Ketac Universal immersed in deionized water, **T** sponsor, and **U** M-150

The surface characteristics of tooth-colored restorative specimens before and after drinks immersion are shown in Fig. 1. Before immersion, the surfaces of both resin composites were relatively smooth on the surface while the glass ionomer restorative materials had a slightly rough surface. After immersion periods, all groups had rougher surfaces. Glass ionomer restorative material groups had the most degraded surfaces (Fig. 1R, U).

## Discussion

The null hypothesis of this study was that surface and color changes of tooth-colored filling materials would not change after cyclic immersion in sport and energy drinks. According to the results obtained from this study, the null hypothesis should be rejected.

The present study was conducted by immersing the specimens in drinks alternately with artificial saliva at room temperature to simulate drinking in everyday life where the saliva washes out the drink in the oral cavity, because the average time for consuming the drink is approximately 2 min [48]. This study simulated this time of 2 min, therefore, approximately 24 cycles of immersion were used. Saliva is also one factor that affects the surface hardness of the material. In addition, temperature also affects degradation [50], therefore, this study was conducted and tested at room temperature to reduce the temperature factor.

In terms of surface hardness, the glass ionomer restorative material had values higher than that of both resin composites. The manufacturer claimed that the properties of this glass ionomer restorative material were improved to have a higher compressive strength and surface hardness, but they did not reveal what additional developments were made. It is expected to be due to improvements in the composition of the powder, which composes of silicon dioxide (SiO_2_) powder of more than 95% by weight. Silicon dioxides are small particles with a wide contact surface that provide good adhesion to the organic polymer, and so provide the material with higher physical properties [2]. Increasing the weight percentage of silicon dioxide also increased the surface hardness of the material [2]. The higher the glass per liquid ratio, the higher the surface hardness. Therefore, the high value of surface hardness of glass cement ionomer in this study might be a result of the properties and type of oxide glass which is a component of the product. The main reactions of glass ionomer restorative materials is acid–base reactions that lead to the formation of a polysalt matrix which is divided into three states; dissolution, gelation, and hardening [3]. The strength of the material increases over time and reaches the highest strength in the first 24 h after mixing, which represents the reaction under the dissolution and gelation state while the hardening state occurs later. This is due to the formation of ionic cross-linking networks and a slow increase in the formation of the silica matrix. The glass matrix formed as a result of the dissolution of glass particles in an acidic solution [4]. As the composition of glass particles (glass) reaches up to 95%, it results in more glass matrices, which increases the surface hardness of the material as well. The surface hardness of the materials were tested with a Vickers hardness tester using a 0.3 µm rectangular-based pyramidal stylus. Eventhough the nanohybrid resin composite, bulk-fill resin composite, and glass ionomer restorative material had an average particles size smaller than the measuring stylus, the glass ionomer restorative material had a larger average particle size than others. The measuring stylus is more likely to press on glass particles resulting in higher surface hardness values than that of nanohybrid resin composite and bulk-fill resin composite materials.

The present results showed that surface hardness values of all groups decreased after immersion in all drinks. The glass ionomer restorative material had significantly the greatest decrease. A plausible reason might arise from the composition of the energy drink which contains citric acid [26]. Citric acid is an acid used to increase the acidity of food and beverages. Its highly corrosive properties are due to its chelating capacity, which binds to calcium from artificial saliva. Therefore, a low pH drink that contains citric acid might have higher erosive capacity properties. Nanohybrid and bulk-fill resin composite had reduced surface hardness values after immersion in the sport and energy drink, even in deionized water. This might be due to the properties of the restorative material, which had highly corroded and dissolved when being under acidic conditions [32]. The acid in the beverage causes the remaining monomers from the reaction to be released from the resin matrix, resulting in a decrease in the surface hardness value [29]. An additional reason may be that the restorative material absorbs water which becomes plasticizing molecules within the resin matrix [30]. As a result, the resin is softer due to the swelling of the polymer network and has reduced friction between polymer chains [31]. Another possible reason might be due to the glass ionomer restorative material containing glass particles mixed in the matrix. In acidic conditions, hydrogen ions from citric acid, an essential component of sport and energy drinks [32], replaces the cations in the matrix that are released from the surface of the material, resulting in a dissolved surface and a decrease in surface hardness values [35].

The increase in surface roughness values of all tooth-colored restorative materials tested in the sport and energy drinks could be a result of the chemical degradation of the restorative material by exposure to chemicals from acidic drinks. It is recognized that the potential in corrosion of materials with acidic conditions depends on the pH acidity [51]. From the present results, the glass ionomer restorative material had a greater increase in surface roughness than that of nanohybrid and bulk-fill resin composite, respectively. Glass ionomer restorative material contains glass particles and a hydrogel matrix [3]. The hydrogen ions from the acid solution are dissolved and replaced by metal cations in the hydrogel matrix of the restorative material. As more positively charged metal ions are displaced, more various ions are drawn from glass particles [3, 52]. This increases the porosity and surface roughness of the restorative material. For resin composite materials, they contain filler particles that are smaller and more homogenous, so there is less space between the inorganic nanoclusters [42]. This results in less surface roughness than glass ionomer restorative materials, which is in agreement with research by Paravina et al. [42]. Materials with different filler particle size, such as hybrid materials, have higher surface roughness and greater color change [42], which corresponds to the results of this study where the bulk-fill resin composite with 0.1–0.004 µm of filler particles had a lower surface roughness value than that of nanohybrid resin composite with 0.4 µm filler particle size. Moreover, resin composites which contain bisphenol A-glycidyl methacrylate (Bis-GMA) or triethyleneglycol dimethacrylate (TEGDMA) increased water absorption from 0 to 1.0 percent by weight according to the proportion of added Bis-GMA or TEGDMA [53]. This makes resin composite resistant to chemical corrosion, having a higher solubility of resin matrix and filler particles. Urethane dimethacrylate (UDMA) exhibits more stain resistance than Bis-GMA due to its water absorption and solubility properties [53], which is consistent with the results of this study. As the nanohybrid resin composite contained TEGDMA, it might result in having a greater surface roughness value than that of bulk-fill resin composite as seen from SEM photomicrographs.

For color change, the glass ionomer restorative materials statistically had greater color value changes than that of both resin composites (*P* < 0.05). Surface roughness caused by erosion and degradation of various chemicals of materials affect the texture of the material, leading to color change from extrinsic factors [43]. In terms of extrinsic factors, it may be caused by pigment agents from food or beverages such as tea, coffee, or from cigarette smoke by adsorption pigments to the surface of the restorative material or by absorption pigments into the resin matrix, affecting the color change. Extrinsic color adhesion is the most important factor affecting the color’s durability and service life of resin composite materials [44]. Sport (Sponsor) and energy drinks (M-150) used in this study contained synthetic colorants as an ingredient. The energy drink contains synthetic dye pigment INS 102 (its generic name is Tartrazine), which is a lemon yellow color pigment, and the synthetic pigment INS 110 (its generic name is Sunset Yellow FCF), which is a yellow-orange color pigment, whereas the sport drink contains just INS 102. These synthetic dyes as well as the low pH of the drinks were important extrinsic factors that could change the color of the restorative materials in this study. This is in agreement with the research by Azer et al. [45] who described that although the colorants in food and beverages are related to extrinsic factors, severity of the color change is also affected by the pH of the drink and foods.

In this study, color change was measured using the CIELAB system which is a system that is recommended to be used to assess color changes, because it can assess even the slightest color change and has advantages in terms of repeatability and sensitivity. From the results of this study, only the glass ionomer restorative material immersed in sport and energy drinks had an ∆E greater than 3.3. The color value change of the material resulted by a change in a* and b* values, which changed its color to red and yellow. This corresponded to the color characteristics of the synthetic colors that are the constituents in both drinks.

Nowadays, although there are many types of tooth-colored restorative materials to choose from and there has been a development to improve the properties, the results of this study revealed that drinking sport and energy drinks affected the surface and color changes of the tooth-colored restorative materials tested. In regard to its clinical relevance, this study proposed that Filtek Bulk Fill Restorative was the most suitable restorative material in patients who consume sport and energy drinks. Although manufacturers of sport and energy drinks claim that drinking such a drink will provide energy and physical health for people who work hard, drinking such drinks might have a detrimental effect on oral and physical health as well.

This study was conducted to simulate drinking sport and energy drinks behavior. However, some limitations are associated with the study which should be taken into account. The cyclic immersion in sport and energy drinks in this study was 14 days, which was not a long time to denote the real drinking of sport and energy drinks in actual life. During actual drinking in a clinical condition, only one side of the restorations is exposed to the oral environment. Whereas the specimens tested immersed in drinks and both sides of the specimens were contacted by the drinks. Regarding the pH and temperature changes in the oral cavity, the properties of the materials might also affect the results of this study as they were not simulated. Moreover, the present study assessed an in vitro condition and no aging process was achieved, whereas other in vivo daily food and drinks can also influence the properties of the restorative materials. Therefore, further studies are required to evaluate the effect of sport and energy drinks in vivo.

## Conclusions

Within the limitations of this study, it was concluded that cyclic immersion in sport and energy drinks statistically affected the surface and color changes of tooth-colored restorative materials by decreasing surface hardness, and increasing surface roughness and color (*P* < 0.05). The energy drink caused a significant decrease in surface hardness and an increase in surface roughness and color when compared to a sport drink (*P* < 0.05). The glass ionomer restorative material immersed in an energy drink was the most degraded material, followed by the nanohybrid and bulk-fill resin composite, respectively.

## Data Availability

The datasets used and/or analyzed during the current study are available from the corresponding author on reasonable request.
